# Estimating evolutionary rates in giant viruses using ancient genomes

**DOI:** 10.1093/ve/vey006

**Published:** 2018-02-27

**Authors:** Sebastián Duchêne, Edward C Holmes

**Affiliations:** 1 Department of Biochemistry and Molecular Biology, Bio21 Molecular Science and Biotechnology Institute, University of Melbourne, Parkville, VIC 3020, Australia; 2 Marie Bashir Institute of Infectious Diseases and Biosecurity, Charles Perkins Centre, School of Life and Environmental Sciences and Sydney Medical School, University of Sydney, Sydney, NSW 2006, Australia

**Keywords:** *Pithovirus*, molecular clock, molecular dating, evolutionary rate, ancient DNA

## Abstract

*Pithovirus sibericum* is a giant (610 Kpb) double-stranded DNA virus discovered in a purportedly 30,000-year-old permafrost sample. A closely related virus, *Pithovirus massiliensis*, was recently isolated from a sewer in southern France. An initial comparison of these two virus genomes assumed that *P. sibericum* was directly ancestral to *P. massiliensis* and gave a maximum evolutionary rate of 2.60 × 10^−5^ nucleotide substitutions per site per year (subs/site/year). If correct, this would make pithoviruses among the fastest-evolving DNA viruses, with rates close to those seen in some RNA viruses. To help determine whether this unusually high rate is accurate we utilized the well-known negative association between evolutionary rate and genome size in DNA microbes. This revealed that a more plausible rate estimate for *Pithovirus* evolution is ∼2.23 × 10^−6^ subs/site/year, with even lower estimates obtained if evolutionary rates are assumed to be time-dependent. Hence, we estimate that *Pithovirus* has evolved at least an order of magnitude more slowly than previously suggested. We then used our new rate estimates to infer a time-scale for *Pithovirus* evolution. Strikingly, this suggests that these viruses could have diverged at least hundreds of thousands of years ago, and hence have evolved over longer time-scales than previously suggested. We propose that the evolutionary rate and time-scale of pithovirus evolution should be reconsidered in the light of these observations and that future estimates of the rate of giant virus evolution should be carefully examined in the context of their biological plausibility.

## 1. Introduction

The discovery of giant viruses has had a major impact on our understanding of the fundamental characteristics of viruses and of their evolution (Raoult and Forterre 2008). Arguably the most striking discovery was that of Mimivirus—a double-stranded DNA (dsDNA) genome of 1.8 Mbp—isolated from an amoeba (*Acathoamoeba* spp.) in 2004 (Raoult et al. 2004). The genome of mimivirus is several fold larger than any previously described dsDNA virus and within the range seen for bacteria. More recently, a broad diversity of DNA viruses with similarly large genomes have been described (Colson, La Scola, and Raoult 2017; Colson, La Scola, et al. 2017), specifically; *Marseillevirus* (350–400 Kbp), *Mollivirus* (652 Kbp), *Faustovirus* (456–491 Kbp), *Kaumoenavirus* (351 Kbp), *Cedratvirus* (575 Kbp), *Pacmanvirus* (395 Kbp), *Pandoravirus* (2, 474 Kbp), and *Pithovirus* (around 640 Kbp).

The first *Pithovirus* described, *Pithovirus sibericum*, was isolated from a supposed 30,000-year-old Siberian permafrost sample inoculated on the amoeba *Acanthamoeba castellanii* (Legendre et al. 2014). This age, and that it was the only known *Pithovirus*, appeared to suggest that *P. sibericum* was an extinct virus lineage. However, in 2016 a closely related isolate, *Pithovirusmassiliensis*, was discovered in a sewage sample taken in southern France (Levasseur et al. 2016), and that exhibited strong sequence similarity to the ancient genome. A comparison of the genomes of *P. masiliensis* and *P. sibericum* also enabled an estimation of the rate of evolutionary change in pithoviruses. In particular, by assuming that *P. sibericum* is the direct ancestor of *P. massiliensis* and that these two viruses have been diverging for 30,000 years, Levasseur *et al.* (2016) estimated a maximum genome-wide evolutionary rate of 2.60 × 10^−5^ nucleotide substitutions per site per year (subs/site/year). This rate estimate is among the highest reported for a dsDNA virus, even compared with those that have much smaller genomes. For example, varicella zoster virus has a genome of 124,884 bp and an estimated evolutionary rate of 6.26 × 10^−6^ subs/site/year, while variola virus (the agent of smallpox) has a genome of 185,578 bp and an estimated mean rate of ∼8.5 × 10^−6^ subs/site/year (Duggan et al. 2016). Most notably, the estimated rate of *Pithovirus* evolution even falls within the range of evolutionary rates observed in some RNA viruses (Duchêne, Holmes, and Ho 2014; Holmes et al. 2016), implying that it is anomalously high.

## 2. Genome size and sampling time shape rates of evolutionary change in microbial populations

One of the most profound observations in studies of microbial populations is that there is a general negative association between rates of evolutionary change and the genome size of the taxa in question (Drake 1991; Gago et al. 2009). This striking pattern has been demonstrated in both RNA and DNA viruses (Sanjuán 2012), and in bacteria (Ochman and Wilson 1987; Duchêne et al. 2016), and has been attributed to the impact of mutational load. Accordingly, if two genomes have very different sizes but the same evolutionary rate, the larger genome will tend to accumulate more mutations, and if most of these mutations are deleterious then the larger genome will in turn have a lower fitness. Therefore, genome size would impose an upper limit on the rate of microbial evolution. In light of the large genome size of *Pithovirus* (∼640 Kbp), the high evolutionary rate estimated by Levasseur et al. would appear to violate this expectation.

**Figure 1.**
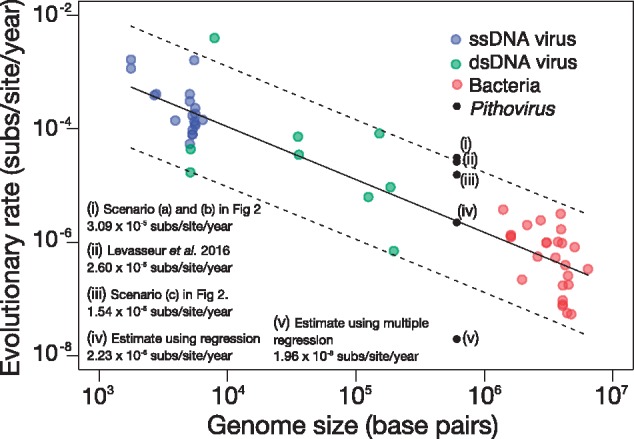
Evolutionary rate estimates for DNA microbes as a function of genome size. The solid line is the least-squares regression and the dashed lines represent the 95% CIs. Each point corresponds to an independent estimate and they are coloured according to the microbe (blue for ssDNA viruses, green for dsDNA viruses, red for bacteria). The black points denote rate estimates for comparisons of *P. sibericum* and *P. massiliensis*, with an average genome size of 646,500 bp: (i) was estimated assuming direct ancestry or immediate divergence between *P. sibericum* and *P. massiliensis* (Scenarios (a) and (b) in Fig. 2); (ii) was estimated by Levasseur et al. (2016); (iii) was estimated assuming that the two viruses are contemporary (Scenario (c) in Fig. 2); (iv) was inferred using a regression of the rate as a function of genome size, log_10_(rate) = −0.93 × log_10_(genome size) – 0.24; and (v) was inferred by fitting a multiple regression of the rate as a function of genome size and sampling time for our bacterial and virus rate estimates, with all variables on a log_10_ scale. The resulting regression followed the equation log_10_(rate) = −0.88 × log_10_(genome size) – 0.68 × log_10_(sampling time) + 0.44, where the sampling time was assumed to be 30,000 years.

To determine whether the evolutionary rate in *Pithovirus* is indeed exceptionally high, we collected a set of published rate estimates for single-stranded (ss) DNA and dsDNA viruses (Sanjuán 2012) and bacteria (Duchêne et al. 2016). We then fitted a linear regression of the rate estimates as a function of genome size, with both variables in a log_10_ scale (Fig. 1). According to this function, we can interpolate the most likely evolutionary rate for *Pithovirus* given its genome size, which is between those of the dsDNA viruses and bacteria sampled here (i.e. *P. sibericum* has a genome of 610 Kpb whereas that of *P. massiliensis* is 683 Kpb, such that we consider the mean genome size of 646.5 Kpb). From this we find that an evolutionary rate of 2.23 × 10^−6^ subs/site/year is the most likely for *Pithovirus*, shown in point (iv) in Fig. 1. The estimate from Levasseur et al. is over an order of magnitude higher, corresponding to the higher range of the confidence interval of the regression, as shown in point (ii) in Fig. 1. Although Levasseur et al. note that their estimate of 2.60 × 10^−5^ subs/site/year is a ‘maximum’ value, we consider it to be implausible given the size of the *Pithovirus* genome.

Estimates of rates of microbial evolution are also impacted by the time-scale of measurement (Duchêne, Holmes, and Ho 2014, Duchêne et al. 2016; Aiewsakun and Katzourakis 2016). In particular, samples collected over a very short time-scale, such as during transmission chains, yield higher rates of evolution than those obtained for samples collected over many years. This pattern has been attributed to a combination of mutational saturation, purifying selection, and substitution model inaccuracy (Duchêne, Holmes, and Ho 2014, Duchêne, Ho, and Holmes 2015). We considered this factor by conducting a multiple linear regression of the evolutionary rate as a function of genome size and sampling time in viral and bacterial microbes as suggested by previous studies (Sanjuan 2012; Duchêne, Holmes, and Ho 2014, Duchêne et al. 2016), with all variables on a log_10_ scale (Fig. 1). By extrapolating the genome size of *Pithovirus* and the age of the permafrost sample (30,000 years) we obtained a rate estimate of 1.96 × 10^−8^ subs/site/year, which is considerably lower than our estimate assuming an association between evolutionary rate and genome size alone. Indeed, this rate estimate is lower than those for bacteria with much larger genomes, and occurs because the sampling time of *Pithovirus* is an order of magnitude older than those of the bacterial data sets included here. As such, this estimate should be interpreted with great caution as it is contingent on the sampling time of *Pithovirus* truly reflecting the age of the permafrost sample, and because it is based on considerable extrapolation.

## 3. Difficulties in estimating the evolutionary rate and time-scale of *Pithovirus*

**Figure 2.**
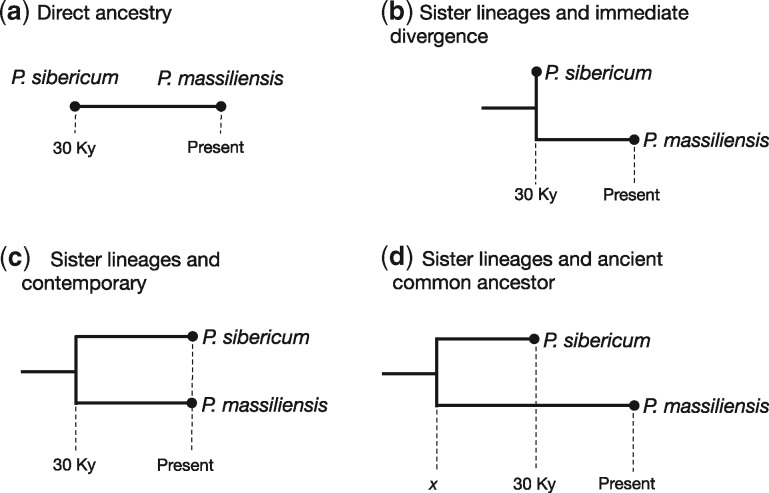
Phylogenetic trees representing four scenarios to estimate the evolutionary rate of *P. sibericum* and *P. massiliensis*. The branch lengths correspond to time and the circles are the two virus samples. The dashed vertical lines denote time points in the tree. In (a) *P. sibericum* is 30,000 years old (Ky) and it is the direct ancestor of *P. massiliensis*. In (b) *P. sibericum* is 30 Ky old and it is the sister-group to *P. massiliensis*, but their divergence occurred immediately after *P. sibericum* was frozen. (c) Shows a scenario in which *P. sibericum* is a contemporary sister taxon of *P. massiliensis*, and their last common ancestor is 30 Ky old. In (d) we consider that *P. sibericum* is a 30 Ky old sister taxon of *P. massiliensis*, and their last common ancestor existed at an unknown time *x*, which we can estimated using the regression of evolutionary rate as a function of genome size in Fig. 1.

We attempted to estimate both the rate and time-scale of *Pithovirus* evolution under a variety of phylogenetic scenarios and involving pairwise comparisons of *Pithovirus* genomes. Although it is difficult to assess the accuracy of these estimates because they are based on only two sequences, they illustrate the range of values that we would expect for the evolutionary rate of *Pithovirus*. These different scenarios are shown in Fig. 2: (a) direct ancestry, in which *P. sibericum* is the direct ancestor of *P. massiliensis* (i.e. as assumed by Levasseur et al.); (b) sister lineages and immediate divergence, in which *P. sibericum* and *P. massiliensis* are sister taxa but diverged immediately after *P. sibericum* was frozen; (c) the two viruses are contemporaneous and shared a common ancestor 30,000 years ago; (d) the two viruses are sister taxa, *P. sibericum* has an age of 30,000 years, and they share a common ancestor at time *x*, where *x* is at least 30,000 years before present. For the purpose of these rate calculations, Scenarios (a) and (b) are equivalent. To conduct our analyses we downloaded the available sequences of *P. sibericum* and *P. massiliensis* from GenBank (accession numbers: KF740664 and FLUR01000008.1; available online at github.com/sebastianduchene/ancient_viruses). Unfortunately, the genome of *P. sibericum* is currently only available as scaffolds and the genomes of the two viruses are not collinear. For this reason we used a single scaffold of *P. massiliensis* that most closely matched the genome of *P. sibericum* according to BLAST E-scores. We then aligned the sequences using MUSCLE v3.8 (Edgar 2004), and used GBlocks (Talavera and Castresana 2007) to remove gaps and regions that were unreliably aligned. Our resulting alignment comprised 128,351 bp, with 40% variable sites.

To estimate the evolutionary distance between the two samples we employed the K80 nucleotide substitution model with a gamma (Γ) distribution of among-site rate variation, enabling us to account for multiple substitutions and the bias of transition over transversion substitutions. This resulted in an evolutionary distance of 0.92 subs/site. For Scenarios (a) and (b) we estimated the evolutionary rate as the evolutionary distance divided by the time to the last common ancestor; that is, 0.92 subs/site/30,000 years = 3.09 × 10^−5^ subs/site/year, which is very similar to the rate reported by Levasseur et al. In Scenario (c) we considered the evolutionary distance divided by two and divided by the time to the last common ancestor; 0.92/2 subs/site/30,000 years = 1.54 × 10^−5^ subs/site/year. Notably, these rate estimates and that reported by Levasseur et al. are within the higher values expected under the relationship between the evolutionary rate and genome size (Fig. 1).

Although Scenario (d) is the most realistic, the time to the last common ancestor (*x* in Fig. 2) is unknown and cannot be inferred simultaneously with the rate. Hence, we considered the most likely rate estimated from the regression of the rate as a function of genome size as a molecular clock calibration, and estimated the time to the last common ancestor. To do this we calculated the evolutionary distance divided by the *Total time* (the length of the path from *x* to *P. sibericum* and from *x* to *P. massiliensis*; Fig. 2), which is equivalent to the evolutionary rate. We then subtract the total time by 30,000 years and divide it by two, to obtain the age of the last common ancestor relative to *P. sibericum*. To obtain the age of the last common ancestor relative to the present we add 30,000 years. This calculation suggested that *P. sibericum* and *P. massiliensis* would have shared a common ancestor about 222,000 years before present (Equation 1). If we consider the much lower rate estimate of 1.96 × 10^−8^ subs/site/year obtained from our multiple regression (i.e. rate as a function of both genome size and sampling time) the last common ancestor would have an age of about 23.5 million years (i.e. substituting 2.23 × 10^−6^ by 1.96 × 10^−8^ in Equation 1).

(1) $$\frac{0.92}{Total\;time}=2.23\times 10^{-6}\;subs/site/year$$

Total time= 414,882.7 years

$$Time\;from\;last\;common\;ancestor\;to\;P.\;sibericum=\frac{\left(414,882.7–30,000\;years\right)}{2}$$

Time of last common ancestor relative to present=Time from x to P. sibericum+30,000

Time of last common ancestor relative to present=222,441.4  years

Estimating evolutionary rates and time-scales in giant viruses is clearly complicated by the paucity of genome data available. Thus, rate estimates must be assessed based on their biological plausibility, including comparisons with evolutionary rates in other DNA viruses. Here, we have utilized the negative association between the evolutionary rate and genome size, which suggests that the evolutionary rate of *Pithovirus* has been previously overestimated by at least an order of magnitude.

A closely related question is whether these viruses originated thousands, hundreds of thousands, or millions of years before present? We postulated an evolutionary scenario in which the age of the common ancestor of *P. sibericum* and *P. massiliensis* is at least 30,000 years old. We consider that this is a far more probable scenario than assuming that *P. sibericum* is directly ancestral to *P. massiliensis*; indeed, just because it is ancient does not necessarily mean that *P. sibericum* has a complete absence of derived substitutions, and it seems highly unlikely that *P. sibericum* would sit exactly at a node on a phylogenetic tree as required if it is truly ancestral to *P. massiliensis*. Following the association between the evolutionary rate and genome size, we find that these viruses may have diverged around 200,000 years before present. If we consider the association of the evolutionary rate, genome size, and sampling time, we obtain a time-scale of millions of years. Both of these estimates suggest a time-scale of *Pithovirus* evolution that is far older than previously envisioned.

Importantly, our evolutionary rate and divergence time estimates are necessarily approximations that assume clock-like behaviour, that mutational saturation is correctly accounted for by the substitution model, that *P. sibericum* is indeed 30,000 years old, and that there is no DNA damage, all of which can affect the accuracy of evolutionary rate estimates (Ho et al. 2015; Duchêne et al. 2016). Indeed, accounting for DNA damage would likely result in an even lower rate and older evolutionary time-scale for these viruses than estimated here, and it is notable that Legendre et al. (2014) did not perform a damage analysis on the *P. sibericum* genomes. Further advances in virus discovery (Li et al. 2015; Shi et al. 2016) are likely to uncover more genetic diversity in these viruses (Halary et al. 2016; Shi, Zhang, and Holmes 2018). This, and the availability of molecular clock calibrating information, will undoubtedly improve our understanding of their evolutionary dynamics.

## Data availability

Sequence alignments and evolutionary rate estimates used in this study are freely available online at: github.com/sebastianduchene/ancient_viruses. The *Pithovirus* sequences used here are also available in GenBank (accession numbers KF740664 and FLUR01000008.1).
